# The Taiji Model of Self II: Developing Self Models and Self-Cultivation Theories Based on the Chinese Cultural Traditions of Taoism and Buddhism

**DOI:** 10.3389/fpsyg.2020.540074

**Published:** 2020-10-14

**Authors:** Zhen-Dong Wang, Feng-Yan Wang

**Affiliations:** ^1^ School of Psychology, Nanjing Normal University, Nanjing, China; ^2^ Institute of Moral Education, Nanjing Normal University, Nanjing, China

**Keywords:** the Taiji Model of Self, self, self-cultivation, Taoism, Buddhism

## Abstract

Based on the construction of the “Taiji Model of Confucian Self” that aims to explain self-structure, the progression of self-cultivation and the dominion of person-making in the context of Chinese Confucian culture, according to the ideas of Taoism and Buddhism, the present study develops the “Taiji Model of Taoist Self” and the “Taiji Model of Buddhist Self” and identifies four realms of Taoist self-cultivation and five realms of Buddhist self-cultivation. In light of the Taiji Model of Taoist Self, self-structure can be divided into the soft self (the Yin part) and the hard self (the Yang part). The Taiji Model of Taoist Self splits the process of self-cultivation into four realms: suren (vulgarian), xianren (solon), shengren (saint), and zhenren (immortal). The Taiji Model of Buddhist Self splits self-structure into the dusty self (the Yin part) and the pure self (the Yang part) and divides the process of self-cultivation into five realms: Humans and Heaven, Arhat, Pratyekabuddha, Bodhisattva, and Buddha.

## Introduction

In recent decades, the construction of models of the self based on cultural context has become a hot topic with the development of cultural psychology (Sedikides and Brewer, 2001; Markus and Kitayama, 2010; Hwang, 2018). Chinese traditional culture, which is based on the ideology systems of Confucianism, Buddhism, and Taoism, has great differences from Western civilization both historically and currently; thus, the Chinese self has an intense cultural specificity (Wu, 2017; Shiah and Hwang, 2019; Wang, 2019). In recent years, although the discussion of the self in different cultural contexts has gradually deepened with the efforts of cultural psychology researchers, self theories and self models still struggle to adequately interpret the self-structure and the process of self-development in the Chinese cultural environment (Yang, 2006; Yang and Lu, 2009; Wang, 2019; Wang et al., 2019).

Because of the close relationship between the construction of the self and the thinking mode in certain cultures (Yang, 2006; Talhelm et al., 2014; Wang, 2018, 2019), Wang et al. (2019) construct the Taiji Model of Confucian Self using the Taiji prototype, which sufficiently reflects the Chinese Yin and Yang thinking mode based on the ontology and epistemology of Chinese Confucianism. Furthermore, they propose the theory of integrated harmony of the self and four realms of Confucian person-making according to different stages of the self-development process.

Confucianism was the mainstream official ideology of China for nearly 2000 years and the most profound ideological system shaping the Chinese self (Feng, 2011, p. 227–230). Although the Taiji Model of Self proposed by Wang et al. (2019) is consistent with the Confucian essence from pre-Qin classical Confucianism (the doctrine of Confucius and Mencius) to Song Ming Confucianism (Neo-Confucianism), the Taoist and Buddhist views of the self are not included in this model. Hence, the model should be called the “Taiji Model of Confucian Self.” On this basis, analyzing the main viewpoints of Taoism and Buddhism regarding the structure and development of the self, we can also construct the “Taiji Model of Taoist Self” and the “Taiji Model of Buddhist Self,” which are isomeric with the Taiji Model of Confucian Self. These models will contribute to a more comprehensive, profound, and accurate understanding of the pluralistic connotations of the Chinese self.

## The Taiji Model of Taoist Self

Because Confucianism and Taoism both praise the thought conveyed in the *I Ching*, which is viewed as the origin of Chinese philosophy, the concepts of Taiji as well as Yin and Yang are included in the Taiji Model of Taoist Self. As a philosophical school with great influence on Chinese history, Taoism has been essential and influential in the emergence and development of the Chinese self. As Joseph Needham said, “Many of the most attractive factors in the personality of Chinese people are derived from Taoist thought. If it was absent in China, the Chinese culture would be like a big tree with rotten roots” (Needham, 1990).

### The Taoist Soft Self and Hard Self

**Figure 1:**
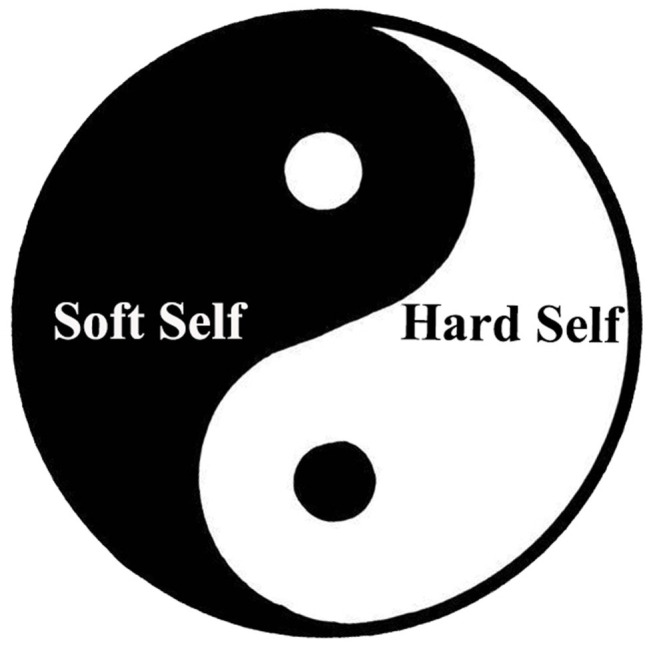
Taiji Model of Taoist Self.

As one of the earliest philosophical schools in China, Taoist thought originated in the spring and autumn periods. Inheriting the thought of the Qian trigram in the I Ching – “As the movement of Heaven is ever vigorous, so must a gentleman untiringly strive along” – Confucians are inclined to advocate strong, firm, and masculine personality characteristics. In contrast, Taoists inherited the thought of the Kun trigram in the I Ching: “Because the condition of the earth is to accept dedication, a gentleman with this character is brought into the outside world” – and they are inclined to advocate clement, tolerant, and feminine personality characteristics. Taoism has always disapproved of Confucians “respecting Yang and degrading Yin” (陽尊陰卑, yang zun yin bei) and has maintained “advocating Yin,” “valuing softness,” and “emphasizing femininity” as the main value orientations (Xu, 1996; Feng, 2011, p. 80–112). Therefore, according to the thought of the Taoist representatives Laozi and Zhuangzi, the Yin and Yang parts of the Taiji Model of Taoist Self can be, respectively, denoted as the “soft” self (柔我, rou wo) and the “hard” self (剛我, gang wo; see Figure 1). The “soft self” represents the traits of the self that reflect softness, weakness, emptiness, simplicity, nondoing, and nature; the “hard self” represents the traits of the self that reflect hardness, fullness, complexity, action, and artificiality.

Many such views can be seen in the *Laozi* (also known as the *Tao Te Ching*); for example, Chapter 43 states, “Gentleness overcomes strength, softness overcomes hardness.” Chapter 19 also states, “People need something they can rely on: highlight your simple self, embrace your original nature, and check your selfishness as you reduce your desires.” Laozi preferred to use water as a metaphor for the characteristics of the soft self. Chapter 8 states, “The best men are like water, which benefits everything but does not compete with it. Water lives in (the bottom-most) places, and it does not like it near the roadways.” Chapter 78 states that “Nothing is weaker than water, but there is nothing better than water in overcoming hardships without alternatives. Therefore, weakness prevails over strength, and softness conquers rigidity.” According to Laozi, Taoists more strongly advocate the soft, feminine, flexible, and liquid self than the hard self, which has strong, masculine, rigid, and solid features. Laozi even extended his proposition to the field of political science and sociology, stating in Chapter 57, “I have done nothing; others may pursue change. I cherish quiet, as it allows one to simply be. I do not engage in business; others may pursue wealth. I have no desire; one should seek to conduct oneself simply and honestly.” In other words, Taoists advocate a soft self with the features of softness, peacefulness, inferiority, nondoing, and desirelessness (Chen, 2009, p. 86–339; Tu and Guo, 2011, 2014).

In addition to the pursuit of being soft and nondoing, the Taoist soft self emphasizes the characteristics of naiveté and nature. This aspect is reflected in the Taoist appraisal of newborn infants, which considers the infant to be the perfect being, in a state of harmonious integration of humanity and heaven (Chen, 2009, p. 269–271, 2016, p. 257). For instance, Chapter 10 of the *Laozi* says, “When you control your vitality to reach a soft state, will you become like a newborn baby?” Chapter 50 says, “Who is rich in character is like an infant?” In the view of Zhuangzi, the authenticity and freedom of the self are consistent with the nature and verity of the Tao. The pursuit of naiveté and the nature of the self carried over into religious Taoism after the Han and Tang Dynasties. “Eliminating factitiousness and retaining naiveté” and “obtaining the authentic self” became the main purposes of Taoist self-cultivation. This progression of self-cultivation and the promotion of the self state was called “xiu zhen” (修真, the cultivation of self-authenticity) by Taoists in later generations (Wang, 1984, p. 3–54).

Correspondingly, in the Taoist view, the “hard self” indicated self part that over-emphasized the secular ethical norms, such as benevolence, and justice, which inherits the strong, firm, and masculine thought of the Qian (乾) diagram from the Book of Changes. Its characteristics were opposite those of the soft self. Chapter 18 of the *Laozi* states that “After the decline of the great Taoism, the doctrine of ‘benevolence’ and ‘justice’ appeared. When knowledge and cleverness appear, what follows is hypocrisy.” Chapter 19 also emphasizes, “When wisdom is banished and knowledge is abandoned, the people will obtain a hundredfold benefit. When ‘benevolence’ is banished and ‘justice’ is abandoned, the people will return to love for their loved ones. When cunning is diminished and ‘utility’ is discarded, thieves and hooligans will disappear… People need what they can rely on: revealing a simple self, embracing one’s original nature, checking one’s selfishness, and reducing desire.” *Zhuangzi, A Protest Against Civilization* even claims, “The loot is located in the thief’s (Confucian) saintly character; he is brave and enterprising and finally shows a knight’s spirit. Here is the wisdom of calculating success and showing kindness in the equal distribution of loot. No robber has ever had these five qualities.” In the Taoist view, the moral principles and the masculine and powerful hard self advocated by Confucianism not only violate the authenticity of the self but also persecute the nature of humanity (Chen, 2009, p. 132–136, 2016, p. 280, 432).

Although the Taoist school in the pre-Qin period had a negative attitude toward the hard self, after the Qin and Han Dynasties, it reconciled with Confucianism. Taoists in the Han Dynasty believed that although the soft self should be advocated and the hard self should be criticized, the former must be restricted, and the latter must be developed within a certain range so that they transform into each other (Liu, 2016, p. 341–459). This is reflected in the dialectical understanding in *Huainanzi*, the Taoist representative magnum opus in the Han Dynasty: “It will be snapped if it is too hard while it will be bent if it is too soft. The hardness comes from softness, and softness can overcome hardness.” Therefore, Taoists after the Han Dynasty mostly contended that the soft self and the hard self function in a manner comparable to the relationship between Yin and Yang in the Taiji figure. That is, the hard self and the soft self do not exist independently; instead, they are inclusive of each other, and the goal of their collaboration is to achieve a combination such that their constituent aspects are essentially the manifestation of the “Tao” (Wang et al., 2019).

### Four Realms of Taoist Self-Cultivation Process

**Figure 2:**
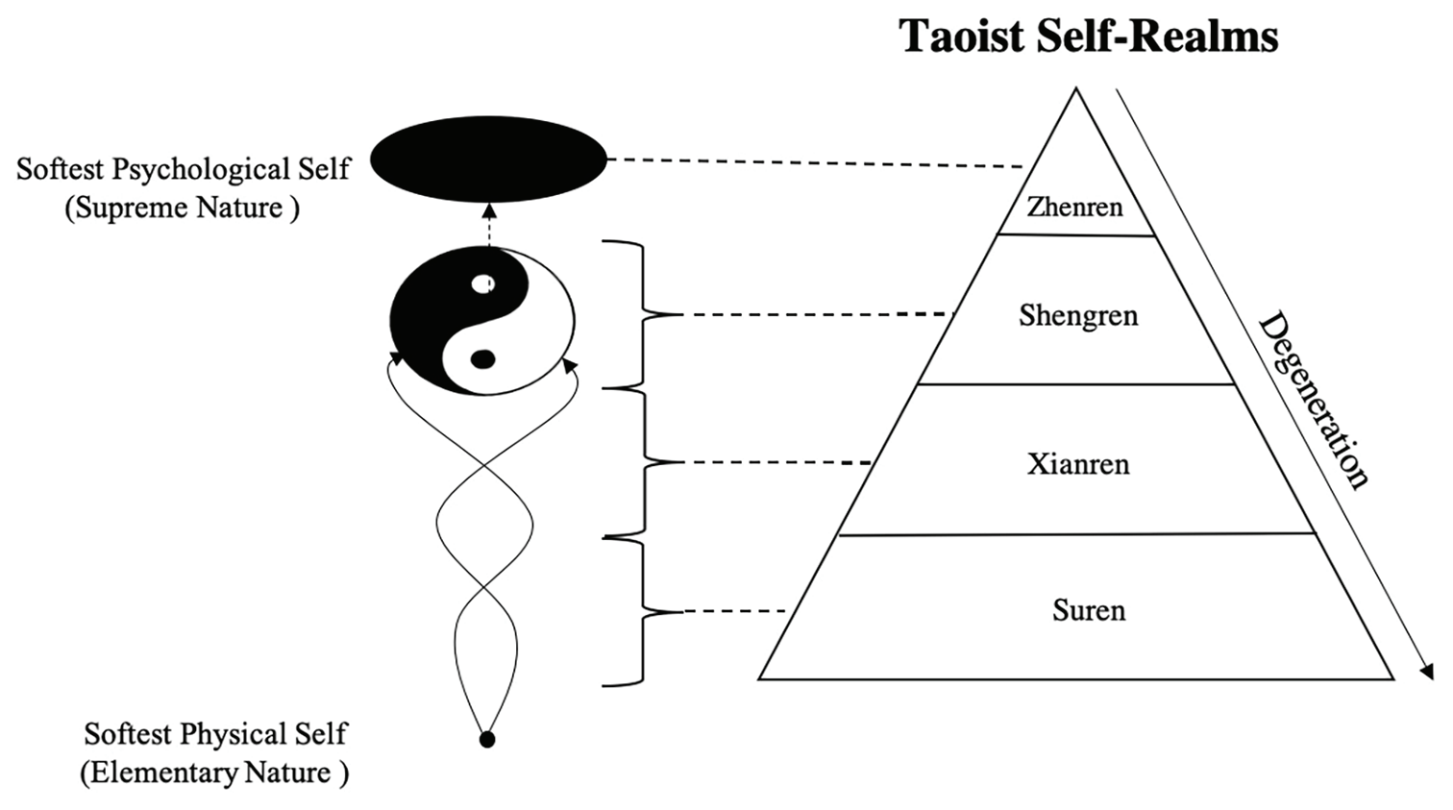
Schematic diagram of Taoist self-realms.

Similar to the Taiji Model of Confucian Self, given the Taoist view of the level of self-cultivation, a stereoscopic Model of Taoist Self can be built with the procedure of Taoist self-advancement shown on the perpendicular axis. Dissimilar states of Taoist self-cultivation can be split corresponding to the diverse states of Taoist self-development. Laozi initially introduces two categories in Taoist self-realm, that is, the shengren (聖人, saint) and the zhongren (眾人, multitude), according to whether one could realize and reach the state of Tao. Based on Laozi’s view, *Zhuangzi, A Happy Excursion* further describes the supreme realms of Taoist person-making: “The zhiren disregards the self; the shenren disregards accomplishment; the shengren disregards reputation” to divide the supreme realms of person-making into three types: zhiren (至人, perfect man), shenren (神人, divine man), and shengren. The Chinese philosophical historian Xu Fu-Guan commented, “The intention of Zhuangzi is to let his own spirit break through his physical body and rise to a place where he can connect himself with the universe.” To realize these three realms is to achieve the pure natural soft self (Xu, 2001, p. 395). However, Zhuangzi’s description is too brief to indicate whether the three realms are paratactic or progressive. Hence, according to Laozi and Zhuangzi, combined with related opinions of the *Inner Canon of Huangdi*, one of the most important works of the Taoist “Huang-Lao School” (Yang, 2015, p. 1–12), the Taoist self-cultivation process can be divided into four realms from bottom to top (see Figure 2): suren (俗人, vulgarian), xianren (賢人, solon), shengren, and zhenren (真人, immortal, including zhiren).

First, suren refers to secular people who do not cultivate themselves according to Taoist doctrine; these people are also known as layfolk or ordinary people. Chapter 20 of the *Laozi* states, “The surens are designing; I am alone and confused. The surens are clever and self-assured; I am alone and depressed… The surens of the world have a purpose; I am stubborn and hypocritical.” That is, although a suren has cleverness in trivial matters through socialization, he/she will not be able to achieve the level of “experiencing the Tao” and will be misled by social norms so that he/she will be far from naiveté and nature (Chen, 2009, p. 137–144).

Second, xianren refers to people who have the ability to recognize the Tao, but the level of recognition is shallow. These people can identify the law and principle of Tao but cannot achieve a nondoing state. *Essential Questions, On Art of Health Cultivation in Ancient Times* states that “there were xianrens who could maintain their health in accordance with the laws of the heavens and the earth, with the variation of the sun or moon and the shifting of the stars; they could comply with the alteration of Yin-Yang and differentiate the four seasons so that they could conduct themselves according to the immortal law of ancient times and prolong their life as long as possible” (Yang, 2015, p. 4).

Third, in the Taoist ideological system, shengren refers to people who abide by the principle of Tao and thus may have the characteristics of softness, weakness, emptiness, simplicity, nondoing, and nature. The *Laozi* Chapter 64 states, “The shengren strives to have no cravings and cherishes goals that are not difficult to attain.” *Zhuangzi, The Way of Heaven* states, “A shengren’s movements are heaven’s movements. His silence is the silence of the earth; he is preoccupied with and rules the whole world. He has no fear of the spirits of the dead. He is not haunted by their souls. His words stem from his emptiness and silence, but they extend to the heavens and the earth and reveal his communication with all things – this is the so-called happiness of heaven.” Thus, Taoist shengrens do not want to influence others through politics as Confucian shengrens do; rather, they aim to reach the realm of heavenly joy through nondoing and a lack of desire (Chen, 2009, p. 178, 296, 2016, p. 367–368).

Fourth, zhenren refers to people who understand the origin of the universe and life thoroughly and who achieve the parts of the self that reflect purity, simplicity, naiveté, and nature in the process of self-cultivation. *Zhuangzi, The Great Supreme* stated, “They are willing to accept life and wait patiently for their ultimate reward. They strive not to misrepresent the Tao, not to supplement nature with human means. Such a person can be called a zhenren. Such people think freely and behave calmly… Living in unrestricted freedom, they can only react naturally to their surroundings. Their tranquility comes from their kindness. In social relations, they maintain their inner character… For them, there is no conflict between humans and God. This is truly a zhenren.” *Dongyuan Ziran Jingjue* said, “The zhenren can embody nothingness and fit the nature of Tao.” That is, the zhenren is the supreme realm of Taoism and is able to fully comprehend and accept the laws of nature, to completely eschew the artificial and achieve pure naiveté and nature and to fully embody the authentic self that is consistent with the Tao. In addition, the “zhiren” mentioned in *Zhuangzi*, *Inner Canon of Huangdi and Tai Ping Jing* can be incorporated into the realm of zhenren, which is at a lower level. *Plain Questions, On Art of Health Cultivation in Ancient Times* states that “there were zhiren, who had superior morals and could master health cultivation, harmonize in Yin-Yang, adapt themselves to the four seasons, abandon worldly interests, and concentrate their spirit, as if they could travel between the heavens and the earth and see or hear beyond eight directions, and this is just the way to extend the life span and become strong and also to attain the zhenren (immortal) status” (Yang, 2015, p. 5–6; Chen, 2016, p. 186; Liu, 2016, p. 35).

As shown in Figure 2, Taoist self-cultivation (the cultivation of self-authenticity) is a process of going from the Elementary Nature, namely, the softest physical self, as infants possessed, and finally reaching the Supreme Nature, i.e., the softest psychological self, which is the state with the Tao. Although the start point and the end point are both nature and soft self, individuals who achieved the realm of zhenren have already experienced the transcendence of “seeing the mountain as the mountain, seeing the mountain not as the mountain, and seeing the mountain still as the mountain,” which is also in line with the aesthetic sentiment pursued by traditional Chinese artists following the Taoist norm (Zhai, 2012, p. 28).

The four realms of Taoist self-development represent the propositions of the Lao-Zhuang Taoist in the pre-Qin period and the Huang-Lao Taoist in the Han Dynasty. These represent the two main schools of Taoism, which reflect the typical Taoist understanding of the self-cultivation stage. At the same time, similar to the theory of the four Confucian realms of self-cultivation, Taoists emphasize the aspiration of and devotion to self-cultivation in accordance with corresponding principles to achieve improvement in the realm of the self (Wang et al., 2019). If one violates the principles of the self-realm that he/she has achieved, he/she will fall back to the lower realm, as Figure 2 shows.

## The Taiji Model of Buddhist Self

Buddhism, which was missionized eastward to China during the Han Dynasty, still had rich Indian features in the early times. Its influence was so slight that many Chinese people regarded it as a part of Taoism before the Jin Dynasty. Its impact gradually grew during the Northern and Southern Dynasties, matured, and then reached its peak in the Sui and Tang Dynasties before eventually evolving into Chinese Buddhism with Chinese cultural characteristics. After the Tang and Song Dynasties, over more than a 1000 years, Buddhist thought has fed back the ontology and epistemology of Confucianism and Taoism. Buddhism has become an important part of Chinese philosophy and has profoundly influenced Chinese traditional culture (Feng, 2011, p. 87–152). Although the Taiji concept and diagram originating from the I Ching were mainly inherited and developed by Confucianism and Taoism, they were also connected with Buddhist thought (Kim, 2010). In this sense, the Taiji Model of Self can also be used to interpret the Buddhist understand of the self.

### The Buddhist Pure Self and Dusty Self

**Figure 3:**
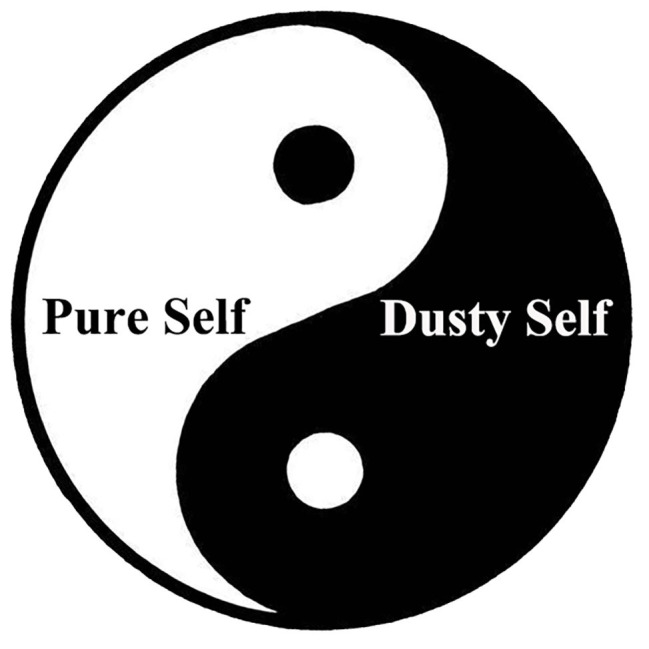
The Taiji Model of Buddhist Self.

Analyzing the Buddhist classics and doctrines reveals that although the Buddhist schools are numerous, their propositions about the self are relatively unified. As Figure 3 shows, in the “Taiji Model of Buddhist Self,” the Yin part can be denoted the “dusty self” (塵我, chen wo), and the Yang part, the “pure self” (淨我, jing wo). The concept of the self in Buddhism originates from the Brahman scriptures, transliterated as “atman,” referring to “an autonomous dominator who does not depend on any conditions and remains constant.” Nirvanasutra said, “If the dharma (phenomena) is substantial, true, constant, autonomous, and independent, with an unchangeable nature, it is called the self (atman).” This means that the self is an eternal, unchangeable, unique, independent, and autonomous substance (Zhang, 1994, p. 197–227).

The dusty self refers to self-clinging (我執, wo zhi) related to the Skandhas (five aggregates of clinging), containing sensations (or feelings, received from form), mental activity or formations, consciousness, form (or material images, impressions), and perceptions, as a constant and autonomous Atman that is the cause of reincarnation. *Samyutta Nikaya* suggested that common people always have an involuntary self-consciousness and feel that there is a unique, constant, and autonomous self in the body. Thus, some of them recognize their own body and mind, personality, and potential as their self; some recognize their social role, status, and reputation as their self, and some recognize their clothing, money, wife, and children as constituting their self. The dusty self is within the scope of Skandhas, obsessed with the Dharma and tainted by the mortal life. It presents as five root Kleshas (annoyance, also known as the five poisons), which are self-ignorance (avidya), self-attachment (raga), self-aversion (dvesha), self-pride (māna), and self-doubt (vicikitsa; Chen, 2006, p. 136–137).

The pure self refers to the notion of the non-ego self (無我, wu wo, anatman or nir-atman) in Buddhism, which is commonly translated as non-self in current Chinese cultural psychological studies (Shiah, 2016). The present article uses “pure self” instead of “non-self” or “non-ego self” to avoid the misunderstanding that the Dharma nature of the self is denied and to indicate that Buddhists advocate cleansing this obsessiveness, viewing the Skandhas as the true self. The use of “no” is to deny the absolute opposite of the two aspects of the matter (such as the presence or absence of the two phases) rather than negating the Dharma nature possessed by the heart and mind (Yang, 1993, p. 312). As Master Huineng indicated, the Great Liberation achieved through enlightenment could result in wisdom that can yield insight into the self-nature of the mind, which is also called the “non-ego” self (Jung, 1969).

Therefore, “this Dharma-door establishes no-thought as its doctrine” and requires that individuals who want to practice Zen bear in mind that the nature of Buddha is in the nature of the self. All people have a dusty mind that covers the nature of Buddha originally present in their self-nature; thus, to cultivate themselves, Buddhists must clear their mind to reveal their Buddha nature. The non-self theory of Buddhism was originally proposed to confront the self-clinging of Sattva (all beings). *Fundamental Verses on the Middle Way, Chapter 18* states that “the non-self will appear after one gets rid of self-clinging.” As *Samyutta Nikaya* recorded, Buddha said that the “non-self is not my self and not my possession; it is the self without the ego.” “Sabbe dhamma anatta” (the doctrine of non-self) is one of three marks of existence (three characteristics of all existence and beings); the other two are impermanence (aniccā) and satisfaction or suffering (dukkha), which are intended to reveal the nonsubstantiality of the phenomenal self. According to the Pawiccasamuppqda (the origin theory) of Buddhism, the most important features of the non-self are “emptiness” and “purity.” Empty is a notion that expresses anitya (impermanence) and the reality of the non-self. Purity refers to the nonpolluted and untroubled nature of reality. Therefore, the non-self can be called the “pure self” (Chen, 2006, p. 139–140; Lou, 2016, p. 91–99).

From the perspective of the Taiji diagram, the dusty self and the pure self also conform with the basic discipline of the transformation of Yin-Yang in Taiji. As there is the seed of the unadulterated self in the dusty self, i.e., Fo Xin (佛心), reflecting the nature of Buddha and the root of wisdom, all living beings have the possibility to become Buddhas. The seed of the dusty self, that is, Fan Xin (凡心), also lies in the pure self, indicating that all beings have instinctive desires. An individual’s dusty self and pure self can transform into each other. If one practices the Dharma and upholds the Buddhist precepts, the dusty self will gradually be cleaned, while if the individual does evil or commits a sin, the pure self will be polluted, thus causing the individual’s degeneration (Zhang, 1994, p. 403–405).

The non-self theory of Buddhism regards pureness as the true nature of the self. Hence, the purpose of Buddhist self-cultivation is to “pure the dusty self and let the pure self emerge.” One will realize the empty and pure nature of self and be able to ward off the annoyances of life and death through this progress. Primitive Buddhism, Theravada Buddhism, and Northern Buddhism all believe that the purpose of self-cultivation is to be aware of the doctrine of non-self and to cease self-clinging (Shiah, 2016; Ding, 2017, p. 2156).

### Five Realms of Buddhist Self-Cultivation Process

**Figure 4:**
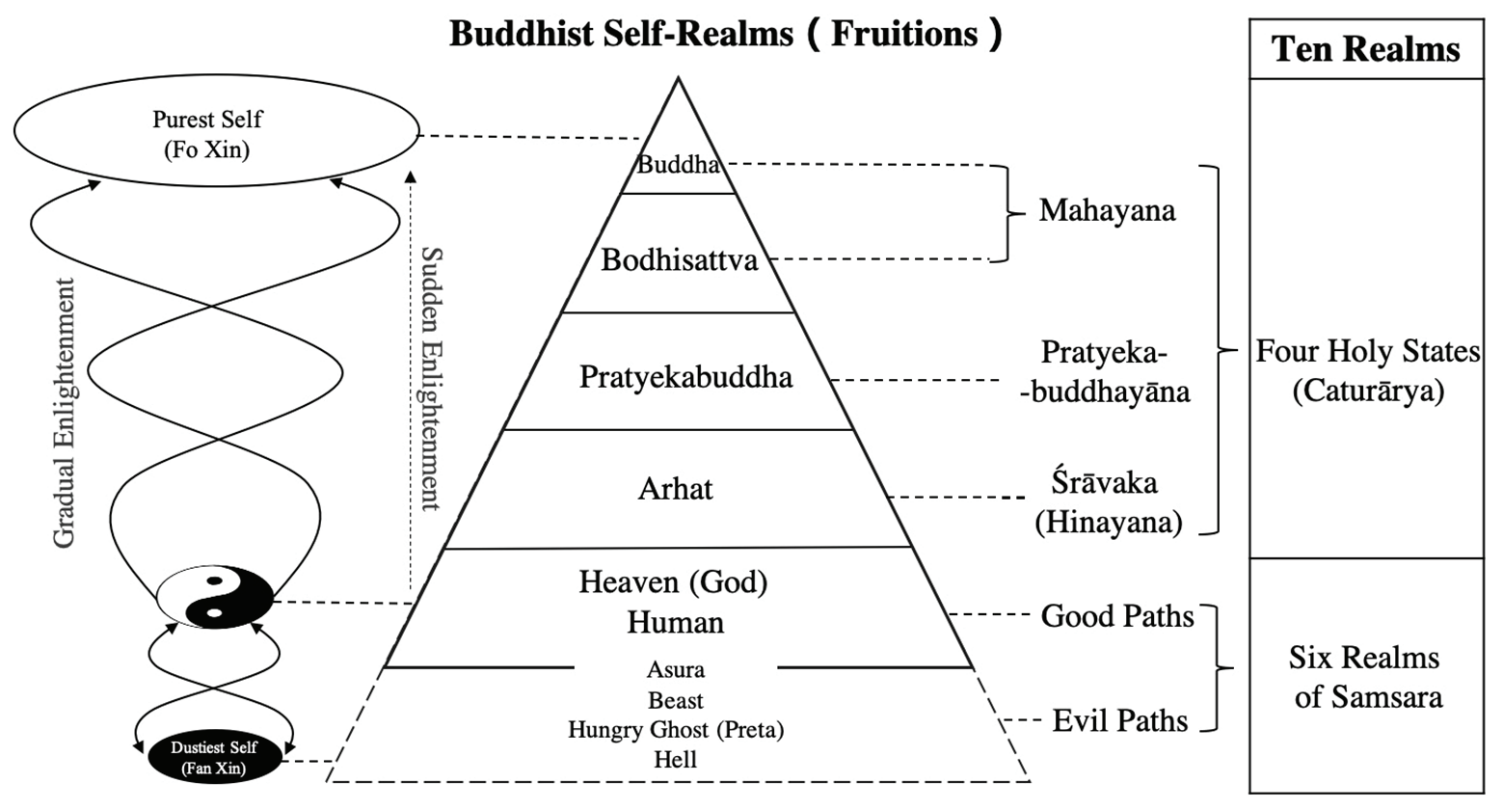
Schematic diagram of Buddhist self-realms (fruitions).

In Buddhism, there is a clear recognition of the ranks of self-cultivation, which is called “fruition” (phala) and refers to the realm achieved by Buddhist practice and the verification of self-achievement. Considering the views of phala in the three vehicles of Buddhism [the three primary Buddhist schools: Mahayana, Pratyekabuddhayāna, and Sāvakayana (also known as Hinayana)], according to the theory of 10 realms/worlds (dasa-dhātavaḥ, 十法界), the realms of Buddhist self-cultivation can be generally divided into the Six Realms of Samara (ṣaḍ-gatīḥ, 六凡) and the Four Holy States (catur-ārya, 四聖). The Six Realms of Samara are the six wheels of cyclic existence of ordinary creatures living in the desire sphere (kāmadhātu, 欲界), form sphere (rūpadhātu, 色界), and formless sphere (ārūpadhātu, 無色界). In accordance with the law of karmic retribution, good intent and deeds contribute to good karma and vice versa (Chen, 2006, p. 95–110; Wang, 2010, p. 51; Hwang, 2018). According to the different conditions of good and evil, the Six Realms of Samara can be divided into Three Evil Paths, including Hell, Hungry Ghosts (Pretas) and Beasts, and Three Good Paths, including Asuras, Humans, and Heaven (devas). Because Asuras are bellicose and aggressive, they are sometimes classified into the Evil Paths, although they have supernatural powers. The Three (or Four) Evil Paths are the worst realms of Reincarnation (Samara, 輪迴); beings in the realm of Humans and Heaven will fall into (rebirth into) them and suffer various miseries and punishments if they perform the corresponding substantive bad deeds (bad karma; Lou, 2016, p. 100–115; Ding, 2017, p. 633). The human realm is the starting point of self-cultivation for an individual involved in the process of person-making. Therefore, as shown in Figure 4, from the bottom to the top, there are five realms: Humans and Heaven, Arhat, Pratyekabuddha, Bodhisattva, and Buddhas.

As long as an individual maintains the five precepts (pañcaśīla) and is committed to abstaining from killing, stealing, sexual misconduct, and lying as well as intoxication, he, she, or it can remain in/be reborn into the human realm. If a human being can practice the 10 Good Karmas and remain still and in deep meditation, he/she will reach the realm of Heaven and be reborn as a deva, which is more powerful, longer lived, and, in general, much happier than humans. However, the living beings in these two realms have not cultivated themselves according to dharma and are not awakened, so they are still trapped in the endless cycle of samsara and cannot break free to reach nirvana (Chen, 2006, p. 96–97; Ding, 2017, p. 268).

If a living being in the Six Realms of Samsara can cultivate himself, herself, or itself according to a religious doctrine, ward off annoyances, and break free from Samsara, then he, she, or it will reach the realms of the Buddhist Holy States. According to the different methods of moksha (emancipation, liberation, and release), as well as the different levels of willpower, the Buddhist Holy States can be divided into Arhat, Pratyekabuddha, Bodhisattva, and Buddha (Chen, 2006, p. 264–272; Wang, 2010, p. 462–464).

From the bottom up, first, Arhat (Sanskrit) or Arahant (Pali) refers to an individual who has gained insight into the true nature of the presence of the Four Noble Truths, that is, dukkha (suffering), samudaya (cause), magga (path), and nirodha (cessation), and has achieved nirvana (Chen, 2006, p. 264–288). According to the Samaññaphala Sutta, there are four stages of enlightenment in Theravada and early Buddhism: Sotāpanna (stream-enterer), Sakadāgāmin (once-returner), nāgāmi (nonreturner), and Arhat. In the view of Chinese Mahayana, these four progressive stages are unified into the Arhat realm, referring to saints who have been freed from samsara (Wang, 2010, p. 4). Although Arhat is the highest fruition that common beings can achieve in Sāvakayana, Arhats can only enlighten themselves to achieve personal freedom and cannot enlighten others. Thus, Arhats are regarded only as “self-enlighteners” at the bottom level of the Four Holy States by Mahayana Buddhists (Ikeda, 2001, p. 100; Ding, 2017, p. 792, 2,843).

The second level of the Four Holy States is Pratyekabuddha, literally “a buddha on their own” or “a private buddha,” which is the highest fruition in Pratyekabuddhayāna (the vehicle of the solitary awakened one). With an exquisite Indriya (spiritual faculty), they can gain wisdom by observing the dependent origination of current affairs by themselves, without supervisors to point out the Dharma (Kumārajīva, 1991; Ding, 2017, p. 2420). Whereas Arhats can only enlighten themselves, Pratyekabuddha lie on a higher self-realm than Arhats. In comparison to full Buddhahood, Pratyekabuddha cannot interpret the Four Noble Truths and cannot bring others to enlightenment. Therefore, in the view of Mahayana, the Pratyekabuddha is the middle level of the Holy States (Wang, 2010, p. 40–48).

Chinese Buddhism mainly developed on the basis of Mahayana Buddhism emphasizes the need to “liberate oneself, liberate others, and liberate all beings.” It claims that everyone has the potential to achieve the same enlightenment as Buddha (Ikeda, 2001, p. 100). Hence, a person who has generated bodhicitta, through a spontaneous desire and compassionate spirit to attain Buddhahood for the benefit of all sentient beings, is called a Bodhisattva. According to *Daśa-bhūmika-vibhāśa-śāstra*, a Bodhisattva can not only enlighten himself or herself but also benefit all sentient beings by teaching them the path of cessation of dukkha; thus, he/she will reach the fruition of Buddha in the future. However, in comparison with Buddha, a Bodhisattva still lacks “Dzogchen” (Great Perfection; Ding, 2017, p. 2111–2117).

The highest level of self-cultivation in Mahayana Buddhism is Buddha, literally meaning “awakened one.” There are three cardinal principles for a Buddha: enlightenment of the self, enlightenment of others, and perfection of enlightened practice. One who achieves all three will reach the realm of Buddha. A Buddha aims for the perfection and liberation of all beings and the attainment of complete enlightenment. Becoming a Buddha is the ultimate goal for every Mahayana Buddhist (Chen, 2016, p. 269–270; Ding, 2017, p. 1152).

According to the view of the change of Yin and Yang in Taiji, the process of Buddhist self-cultivation is the transformation from Fan Xin to Fo Xin. Fo Xin is the heart of enlightenment, referring to the pure self within everyone’s mind, also called Buddha nature or Bodhicitta. *Amitayurdhyana Sutra* states, “The Fo Xin is Great Compassion. It embraces sentient beings with unconditional Benevolence.” In contrast, Fan Xin, also called Kama, refers to the desires, wishes, and longing of sentient beings in the three spheres. These include the five sensual desires of wealth, sex, fame, appetite, and sleep, representing the three unwholesome roots (akuśala-mūla) or three poisons (triviṣa) of Dvesha (aversion), Moha (delusion, confusion), and Raga (greed, sensual attachment). Fan Xin is the reflection of the dusty self polluted by sensual desires and refers to one’s tribulations or character impurities, the derivation of taṇhā (craving), and the cause of dukkha (pain, suffering, dissatisfaction; Chen, 2006, p. 250–253; Wang, 2010, p. 119–121; Ding, 2017, p. 309). *The Treatise on the Great Perfection of Wisdom, Chapter 17* states, “All sentient beings are often annoyed by the five sensual desires, but they are still eager to ask for it… All living creatures are tempted by the five sensual desires, never given up until death, so they have to suffer endless misery in their lives.” Individuals in the six realms of Samsara have both Fo Xin and Fan Xin. With the improvement in Dharma practice and the deepening of the Threefold Training (śikṣā) – the training of virtue, mind, and wisdom – Fo Xin will extend and Fan Xin will gradually shrink (Chen, 2006, p. 64; Ding, 2017, p. 1154–1156).

In contrast to Confucian and Taoist self-cultivation models, in the realm of Buddhist self-cultivation, only sentient beings in the Six Realms of Samsara may experience the reversibility of self-development with a change in karma. Once the realm of Arhat is achieved, there is only a possibility of rising into a higher realm and no possibility of degenerating (see Figure 4).

## Conclusion

In summary, based on traditional Chinese Taoist and Buddhist thought, the present work further develops the Taiji Model of Self and draws the following conclusions.

First, in accordance with Laozi and Zhuangzi’s point of view, in the Taiji Model of Taoist Self, the Yin and Yang parts correspond to the soft self and hard self, respectively. The self-cultivation process advocated by Taoism is oriented toward a soft self with the features of softness, peacefulness, inferiority, nondoing, and desirelessness. Hereby, the realm that can be achieved by Taoist self-cultivation can be divided into four levels from bottom to top: suren, xianren, shengren, and zhenren.

Second, in the Taiji Model of Buddhist Self, the Yin part denotes the dusty self, and the Yang part denotes the pure self. The goal of self-cultivation in Buddhism is to “pure the dusty self and let the pure self emerge” so that the empty and pure nature of the self will be realized. From the starting point of humans, the process of Buddhist self-cultivation can be divided into five realms: Humans and Heaven, Arhat, Pratyekabuddha, Bodhisattva, and Buddha.

Third, the Yin and Yang features contained in the Taiji diagram can be reflected in the interrelationship of the self-structure in the Taiji Model of Taoist Self and the Taiji Model of Buddhist Self, as well as the processes of Taoist and Buddhist self-cultivation.

## Author Contributions

The contribution of Z-DW was in constructing the theory and writing the paper. The contribution of F-YW was in constructing the theory and revising the paper. Both authors contributed to the article and approved the submitted version.

### Conflict of Interest

The authors declare that the research was conducted in the absence of any commercial or financial relationships that could be construed as a potential conflict of interest.
